# Facteurs associés aux circulaires du cordon: étude cas-témoin dans trois hôpitaux de Yaoundé

**DOI:** 10.11604/pamj.2020.35.23.19365

**Published:** 2020-01-27

**Authors:** Florent Ymele Fouelifack, Line Claire Meche Dahda, Jeanne Hortence Fouedjio, Loic Dongmo Fouelifa, Robinson Enow Mbu

**Affiliations:** 1 Hôpital Central de Yaoundé, Yaoundé, Cameroun; 2 Institut Supérieur de Technologie Médicale de Nkolondom, Yaoundé, Cameroun; 3 Groupe Associatif pour la Recherche, l’Education et la Santé (GARES-Falaise), Dschang, Cameroun; 4 Faculté de Médicine et des Sciences Biomédicales de l’Université de Yaoundé I, Yaoundé, Cameroun; 5 Faculté des Sciences de la Santé, Université de Lomé, Ecole des Services de Santé des Armées de Lomé, Lomé, Togo; 6 Ministère de la Santé Publique, Yaoundé, Cameroun

**Keywords:** Facteurs associés, circulaire du cordon, Yaoundé, Factors associated, coiling of umbilical cord, Yaoundé

## Abstract

**Introduction:**

La circulaire du cordon ombilical correspond à l’enroulement du cordon ombilical en un ou plusieurs tours sur une partie du fœtus. Anomalie la plus fréquente du cordon, sa prévalence varie selon les auteurs de 5,7% à 35,1%. En 2011, le taux de mortalité périnatale liée à la circulaire du cordon au Cameroun était de 6,1%. Cependant ses facteurs associés restent peu connus dans notre contexte. Notre objectif était de déterminer les facteurs associés aux circulaires du cordon dans trois hôpitaux de Yaoundé

**Méthodes:**

Il s’agissait d’une étude analytique de type cas-témoin, réalisée pendant 4 mois dans les maternités de l’Hôpital Central de Yaoundé, du Centre Hospitalier et Universitaire de Yaoundé et du Centre d’Animation Sociale et Sanitaire de Nkoldongo. Pour un cas (nouveau-nés avec circulaire du cordon), on recrutait 2 témoins (nouveau-nés sans circulaire) tous en présentation céphalique, issus de grossesses monofoetales à terme. Les données recueillies étaient compilées sur des fiches techniques préétablies, saisies et analysées grâce aux logiciels Microsoft Excel 2017 et SPSS version 23. Les outils utilisés pour l’analyse étaient la moyenne d’âge, l’écart type et la fréquence, le rapport de cote cru (OR) et/ou ajusté (aOR) avec leur intervalle de confiance à 95%. P était considéré significatif pour toute valeur inférieure à 5%

**Résultats:**

Sur un total de 3300 accouchements, 500 nouveau-nés soit 15,15% avaient une circulaire du cordon. Toutes les circulaires étaient autour du cou. Nous avons retenu et analysé 136 nouveau-nés avec circulaire du cordon (cas) pour 272 nouveau-nés sans circulaire (témoins). Les facteurs indépendamment associés aux circulaires étaient non modifiables: longueur du cordon = 70cm (ORa = 32 IC = 17,5-35 P = 0,02), sexe masculin (ORa = 67,09 IC = 22,31 - 97,46 P = 0,001), APGAR 5^ème^ minute < 7 (ORa = 76,98 IC =2,19-27,05 P = 0,017), et modifiables: l’âge gestationnel ≥ 42SA (ORa = 15,15 IC = 6,14-18,2 P=0,001)

**Conclusion:**

La circulaire du cordon est une anomalie fréquente du cordon. Nous suggérons aux décideurs de sensibiliser davantage le personnel de santé et la population sur l’importance de l’échographie du troisième trimestre afin de rechercher et prévoir la prise en charge des nouveau-nés avec circulaire du cordon. Les cliniciens devraient éviter autant que possible le post terme.

## Abstract (en)

**Introduction:**

The coiling of umbilical cord is the winding of the umbilical cord around a part of the fetal body once or several times. It is the most common abnormality of the cord, its prevalence varies according to the authors from 5.7% to 35.1%. In 2011, the rate of perinatal mortality due to the coiling of umbilical cord in Cameroun was 6.1%. However factors associated to it are little known in our context. Our purpose was to determine factors associated to the coiling of umbilical cord in three hospitals in Yaoundé

**Methods:**

We conducted an analytical case-control study in the Maternity Unit at the Central Hospital of Yaoundé, the University Teaching Hospital of Yaoundé and the Social and Health Nkoldongo Animation Center over a period of 4 months. The study-group composed of newborns with coiling of umbilical cord was compared with two control groups (newborns without coiling of umbilical cord). All the fetuses were cephalic at delivery (singleton pregnancies at term). Pre-established technical data sheets were filled with data collected and analyzed based on the Microsoft Excel 2017 and SPSS software Version 23. The parameters used for the analysis were the average age, standard deviation and frequency, the raw odds ratio (OR) and/or adjusted (aOR) with their 95% confidence interval. P was considered significant for any value less than 5%

**Results:**

Out of a total of 3,300 deliveries, 500 newborns (15.15%) had coiling of umbilical cord. All the coils were around the neck. We retained and studied 136 newborns with coiling of umbilical cord (study group) vs 272 newborns without coiling of umbilical cord (control group). Factors independently associated with coiling were non editable: length of the cord = 70cm (ORa = 32 CI = 17.5-35 p = 0.02), male sex (ORa = 67.09 CI = 22.31 - 97.46 P = 0.001), APGAR score 5th minute <7 (ORa =76.98 CI = 2.19 - 27.05 P=0.017) and modifiable factors were gestational age = 42WA (ORa = 15.15 CI = 6.14-18.2 P = 0.001)

**Conclusion:**

The coiling of umbilical cord is a frequent cord abnormality. We suggest to the decision-makers to increase awareness among health workers and the population on the importance of ultrasound scan of the third trimester in order to detect coiling of umbilical cord and implement appropriate manage newborns. Clinicians should avoid as much as possible post-term pregnancy.

## Introduction

La circulaire du cordon ombilical correspond à l’enroulement du cordon ombilical en un ou plusieurs tours sur une partie du fœtus. Généralement considérée comme bénigne [1], elle représente l’anomalie la plus fréquente du cordon et peut affecter toutes les grossesses [2]. Sa prévalence varie de 5,7% en Inde [3] à 35,1% en Suisse [4]. Au Cameroun, elle varie selon les études de 5,5 [5] à 16,2% [6]. Son incidence augmenterait avec l’âge gestationnel passant ainsi de 5,8% à la 20^ème^ semaine à 29% à la 42^ème^ semaine d’aménorrhée [7].

Le diagnostic anténatal de la circulaire du cordon est surtout basé sur l’échographie. La sensibilité de l’échographie bidimensionnelle pour son diagnostic est de 69% tandis que celle de l’échographie doppler couleur varie entre 83 à 97% [8]. Si pour certains auteurs la circulaire du cordon n’entraine généralement pas de complications néonatales critiques [6, 9], pour d’autres, il peut avoir un impact majeur sur le nouveau-né et entrainer une asphyxie néonatale se traduisant par un mauvais score d’Apgar. Nkwabong *et al*. [5] et Foumane *et al.* [10] retrouvaient respectivement les taux de mortalités périnatale de 6,1% en 2011 et néonatale de 6,8% en 2013, liés à la circulaire du cordon. Il ressort de cette controverse, que la circulaire du cordon n’est pas toujours anodine. La recherche des facteurs associés aux circulaires du cordon pourrait nous permettre de faire un diagnostic précoce et par conséquent d’améliorer le pronostic périnatal, raison pour laquelle nous avons entrepris cette étude dont l’objectif général était de rechercher les facteurs associés aux circulaires du cordon.

## Méthodes

### Type, lieux, période et durée de l’étude

Nous avons mené une étude analytique de type cas-témoins dans 3 formations hospitalières de la ville de Yaoundé, du 1er février au 30 juin 2017, soit sur une durée de 5 mois. Il s’agissait des maternités des Services de Gynécologie et d’Obstétrique du Centre Hospitalier et Universitaire (CHU), de l’Hôpital Central de Yaoundé (HCY) et du Centre d’Animation Social et Sanitaire (CASS) de Nkoldongo. Ces formations hospitalières réalisent chacun un nombre élevé d’accouchements, ce qui nous permettait d’atteindre facilement la taille minimale de notre échantillon.

### Echantillonnage

L’échantillonnage était consécutif. L’étude portait sur les bébés nés pendant la période d’étude présentant ou non au moins un tour de circulaire du cordon à la naissance. Etaient inclus tous les nouveau-nés en présentation céphalique, issu de grossesses monofoetales et à terme. Etaient exclus tous les nouveau-nés dont la maman refusait le consentement ou le retirait au cours de l’étude. La taille minimale de chaque groupe était calculée en utilisant la formule de Schlesselman suivante:

N=2(Za+Zβ)2×P(1−P)/(Po−P1)2

Où N = taille de l’échantillon, P = prévalence = (P1+P2)/2. Pour un intervalle de confiance à 95% on aura un risque a = 5%; Za = constante = 1,96 = prévalence des complications liées aux circulaires du cordon = 50% = 0,5 = 25% = 0,25. Après application numérique, N = 79 cas. Mais pour augmenter la validité de nos résultats, nous avons recruté le maximum de cas qui respectaient nos critères de sélection, soit 136 cas (nouveau-nés avec circulaire du cordon) et 272 témoins (nouveau-nés sans circulaire du cordon).

### Procédure

Dès l’arrivée des parturientes, un counseling était fait pour les informer de l’objectif de l’étude. Leur consentement verbal ou écrit était requis avant le début de l’interrogatoire. Pour tout nouveau-né sélectionné, les antécédents, l’histoire de la grossesse, les caractéristiques du travail ayant conduit à son accouchement, et les éventuelles complications immédiates étaient recherchés. Les renseignements reçus lors de l’interrogatoire étaient complétés à partir des registres et les carnets de consultation et consignés sur une fiche technique anonyme préétablie et testée. Pour tout nouveau-né présentant une circulaire du cordon (cas), 2 nouveau-nés accouchés immédiatement ne présentant pas de circulaire (témoins) étaient recrutés. Toutes ces informations étaient regroupées en 3 grands groupes de variables à analyser: caractéristiques sociodémographiques, caractéristiques obstétricales et caractéristiques fœtales et funiculaires. Les caractéristiques sociodémographiques des parturientes: l’âge (en années); la profession (ménagère, élève/étudiante, fonctionnaire, commerçante et autres); le statut matrimonial (marié, célibataire); la région d’origine; la religion (chrétienne, musulmane). Les caractéristiques obstétricales telles que la parité (nombre d’accouchement à terme, divisé en primipare (premier accouchement), multipare (nombre d’accouchement supérieur à un et inférieur à 6) et grande multipare (nombre d’accouchement supérieur ou égal à 6); l’âge gestationnel calculé à partir de la date des dernières règles et confirmé par une échographie du premier trimestre (à < 20 SA), l’hypotrophie, l’oligoamnios, l’hydramnios, la dilatation à l’arrivée en centimètres, anomalies du rythme cardiaque fœtal: normal (120-160 battements par minutes), tachycardie (> 160 battements par minutes), bradycardie (< 120 battements par minutes), absences de bruits du cœur fœtal (pas de battement), la durée du deuxième stade du travail en minutes (ou phase d’expulsion): elle correspond au temps écoulé entre la dilatation complète du col utérin (10cm) et la sortie du fœtus et ne concernait que les accouchements par voie basse. La couleur du liquide amniotique était classée en clair, verdâtre (méconiale), jaunâtre, hémorragique. Le type de circulaire du cordon était en fonction de l’étroitesse de l’enroulement, dit « serré » lorsqu’il était impossible de passer l’index entre le cordon et le cou du nouveau-né, et « lâche » dans le cas contraire. On notait le nombre de tours du cordon autour du cou du nouveau-né, la présence ou non de nœuds du cordon, la longueur du cordon en cm, le score d’Apgar à la 5^ème^ minute, le poids de naissance en grammes et le sexe du nouveau-né.

### Collecte et analyse des données

Les données recueillies étaient consignées sur des fiches techniques préétablies et testées, saisies grâce au logiciel Excel 2013 et analysées grâce aux logiciels Microsoft Excel 2013 et SPSS version 23. Les outils utilisés pour l’analyse descriptive étaient la moyenne d’âge, l’écart type et la fréquence. L’association entre les variables étudiées et la circulaire du cordon était recherchée à l’aide du rapport de cote (Odd ratio) cru et/ou ajusté, avec son intervalle de confiance à 95%. P était considéré comme significatif pour toute valeur inférieure à 5%.

## Résultats

### Fréquence de la circulaire du cordon

Durant la période de l’étude 3300 nouveau-nés issus des grossesses mono fœtales étaient accouchés, parmi lesquels 500 nouveau-nés avec circulaire du cordon soit une fréquence de circulaires du cordon de 15,15%. Toutes les circulaires étaient autour du cou du fœtus. Sur les 3300 nouveau-nés, nous en avons recruté au total 408 nouveau-nés soit 136 cas contre 272 témoins. Parmi les 136 nouveau-nés avec circulaire du cordon, 103 avaient les circulaires lâches et 33 les circulaires serrés. Quant au nombre de tours du cordon, on notait 106 nouveau-nés avec 1 tour, 27 avaient 2 tours et 3 avaient 3 tours.

### Caractéristiques sociodémographiques et circulaire du cordon

La recherche d’association entre les caractéristiques sociodémographiques des accouchées et la survenue de la circulaire du cordon est représentée dans le Tableau 1. Nous n’avons retrouvé aucune association significative entre l’âge maternel, le statut matrimonial, la religion, la profession des parturientes et la survenue de la circulaire du cordon.

**Tableau 1:** Association entre les caractéristiques sociodémographiques des accouchées et la survenue de circulaire du cordon

Variable	Cas		Témoins		OR	IC à 95%		P
	N	(%)	N	(%)		Min	max	
**Age gestationnel (en tranches de 5 semaines)**								
[15-20[	15	11,0	20	7,4	1,50	0,77	2,93	0,300
[20-25[	27	19,9	61	22,4	0,89	0,56	1,39	0,500
[25-30[	49	36,0	85	36,0	1,15	0,81	1,64	0,499
[30-35[	27	19,9	77	28,3	0,70	0,45	1,09	0,500
[35-40[	15	11,0	26	9,6	1,15	0,61	2,18	0,455
≥40	3	2,2	3	1,1	2,00	0,40	9,91	0,410
Total	136	100,0	272	100,0				
**Statut matrimonial**								
Mariée	49	36,0	63	23,2	1,56	1,07	2,26	0,7
Célibataire	87	64,0	209	76,8	0,83	0,65	1,07	0,27
Total	136	100,0	272	100,0				
**Religion**								
Chrétienne	130	95,6	266	97,8	0,98	0,79	1,21	0,879
Musulmane	6	4,4	6	2,2	2,00	0,65	6,20	0,237
Total	136	100,0	272	100,0				
**Profession**								
Ménagère	48	35,3	87	32,0	1,10	0,78	1,57	0,499
Elève/Etudiante	33	24,3	67	24,6	0,99	0,65	1,49	0,875
Fonctionnaire	24	17,6	58	21,3	0,83	0,51	1,33	0,500
Autres	31	22,8	60	22,1	1,03	0,67	1,59	0,912
Total	136	100,0	272	100,0				

### Profil obstétrical des accouchées

La recherche d’association entre les caractéristiques obstétricales et la survenue de la circulaire du cordon est représentée dans le Tableau 2. Le post terme (≥ 42 SA), les anomalies du rythme cardiaque fœtal, la couleur verdâtre du liquide amniotique, et la durée du 2^ème^ stade du travail > 30 minutes étaient des facteurs prédictifs de circulaire du cordon avec respectivement OR = 2,67 (IC =1,37-5,21; P = 0,006), OR = 4,32 (IC = 2,78-6,71; P =< 0,0001), OR = 2,05 (IC = 1,32-3,19; P =< 0,0001) et OR=16,4 (IC=9,6-28,03; P =< 0,0001).

**Tableau 2:** Association entre le profil obstétrical et la survenue de la circulaire du cordon

Variable	Cas		Témoins		OR	IC à 95%		P
	N	(%)	N	(%)		Min	Max	
**Parité**								
Primipare	38	27,9	83	30,5	0,92	0,62	1,34	0,72
Multipare	94	69,1	169	62,1	1,11	0,86	1,43	0,56
Grande multipare	4	2,9	20	7,4	0,40	0,14	1,17	0,13
Total	136	100,0	272	100,0				
**Age gestationnel (en semaines)**								
<42	116	85,3	257	94,5	0,90	0,73	1,12	0,504
≥42	20	14,7	15	5,5	**2,67**	1,37	5,21	**0,006**
Total	136	100,0	272	100,0				
**Anomalie du rythme cardiaque fœtal**								
Oui	80	58,8	37	13,6	**4,32**	2,78	6,71	**< 0,0001**
Non	56	41,2	235	86,4	0,47	0,33	0,68	**< 0,0001**
Total	136	100,0	272	100,0				
**Couleur du liquide amniotique**								
Clair	85	62,5	216	79,4	0,79	0,61	1,01	0,123
Verdâtre	40	29,4	39	14,3	**2,05**	1,32	3,19	**< 0,0001**
Jaunâtre	11	8,1	17	6,3	1,29	0,61	2,76	0,557
Total	136	100,0	272	100,0				
**Durée du 2^ème^ stade du travail (en minutes)**								
**≤**30	13	9,6	257	94,5	0,10	0,06	0,18	**< 0,0001**
>30	123	90,4	15	5,5	**16,40**	9,60	28,03	**< 0,0001**
Total	136	100,0	272	100,0				

### Caractéristiques fœtales et funiculaires

La recherche d’association entre le poids, le sexe du nouveau-né, la longueur du cordon, le nœud du cordon et la survenue de la circulaire du cordon est représentée dans le Tableau 3. Le sexe masculin et la longueur du cordon ombilical ≥ 70cm étaient des facteurs de risque de circulaire du cordon avec respectivement OR = 2,91 (IC = 1,03-3,1; P = 0,009) et OR = 4,4 (IC =2,74-7,06; P = 0,0001).

**Tableau 3:** Association entre le poids, le sexe du nouveau-né, la longueur, le nœud du cordon et la survenue de la circulaire du cordon

Variable	Cas		Témoins		OR	IC à 95%		P
	N	(%)	N	(%)		Min	Max	
**Poids des nouveau-nés (en grammes)**								
<2500	11	8,1	11	4,0	**2,00**	0,87	4,61	0,08
[2500-3500[	82	60,3	198	72,8	0,83	0,64	1,07	0,2727
[3500-4000[	38	27,9	47	17,3	1,62	1,05	2,48	0,126
4000 et plus	5	3,7	16	5,9	0,63	0,23	1,71	0,417
Total	136	100,0	272	100,0				
**Sexe du nouveau-né**								
Masculin	118	86,76	81	29,8	**2,91**	1,03	3,1	**0,009**
Féminin	18	13,24	191	70,2	0,71	0,53	0,97	**0,001**
Total	136	100,0	272	100,0				
**Longueur du cordon en Cm**								
<70	81	59,6	247	90,8	0,66	0,51	0,84	**< 0,0001**
≥70	55	40,4	25	9,2	**4,40**	2,74	7,06	**< 0,0001**
Total	136	100,0	272	100,0				
**Présence du Nœud du cordon**								
Oui	4	2,9	0	0,0				
Non	132	97,1	268	100,0	0,99	0,80	1,21	0,92
Total	136	100,0	268	100,0				

### Régression logistique

Pour rechercher les facteurs indépendamment associés à la circulaire du cordon, une régression logistique était faite en incluant toutes les variables préalablement associées lors des analyses uni variées. Les résultats sont représentés dans le Tableau 4. Les facteurs indépendamment associés au circulaire du cordon étaient: l’âge gestationnel ≥ 42 SA, la longueur du cordon ≥ 70cm, le sexe masculin et le score d’APGAR à la 5*ème* minute < 7.

**Tableau 4:** Régression logistique

Facteurs	OR	IC		OR ajusté	IC à 95%		P
Age gestationnel ≥42 SA	2,67	1,37	5,21	15,15	6,14	18,2	0,001
Anomalie du RCF pendant le travail	4,32	2,78	6,71	10,45	6,05	80,87	0,06
Liquide amniotique verdâtre	2,05	1,32	3,19	1,86	0,58	4,35	0,07
Durée d’expulsion >30minutes	16,4	9,6	28,03	17,2	10,92	30,02	0,08
Longueur du cordon ≥70Cm	4,40	2,74	7,06	32	17,5	35	0,02
Sexe masculin	2,91	1,03	3,1	67,09	22,31	97,46	0,001
APGAR 5^ème^ minute <7	8	1,7	37,67	76,98	2,19	2705,58	0,017

## Discussion

### Facteur limitant dans notre étude

Bien qu’ayant interrogé nous-mêmes les parturientes, nous n’avons pas pu assister à tous les accouchements. Nous n’étions donc pas toujours présents pour mesurer les cordons et les registres n’étaient pas suffisamment remplis pour nous fournir les informations nécessaires. Par conséquent, nous n’avons pas recruté tous les cas de circulaire survenu au cours de notre période d’étude. Cependant nous pensons que nos résultats sont fiables étant donné que la taille minimale de chaque groupe de 79 est largement inférieure à la taille utilisée pour nos analyses.

### Fréquence des circulaires du cordon

Notre fréquence de 15,15% de circulaires du cordon pour les grossesses monofoetales se rapproche de celle de 16,2% retrouvée par Kemfang *et al*. en 2011 à l’Hôpital Général de Yaoundé [6]. Par contre elle est supérieure à 5,5% retrouvée en 2011 par Nkwabong *et al*. [5] et a 8.3% par Foumane *et al.* en 2016 [11].

### Caractéristiques sociodémographiques et obstétricales

Aucune association n’était retrouvée entre l’âge maternel, le statut matrimonial, l’appartenance à un groupe religieux, la profession et le circulaire du cordon (tableau1). Dans son étude, Dippel n’avait trouvé aucune association entre l’âge maternel et la circulaire du cordon [12].

### Profil obstétrical

Nous n’avons pas retrouvé d’association significative entre le nombre d’accouchement (Tableau 2) et la survenue de circulaire du cordon. Ce résultat corrobore avec celui trouvé par Foumane *et al.* dans une étude menée en 2013 à l’Hôpital Gynéco-Obstétrique et Pédiatrique de Yaoundé où ils n’ont retrouvé aucune association entre la circulaire du cordon et la parité [10]. Par contre, Rhoades *et al*. [13] avaient retrouvé la primiparité comme facteur de risque de circulaire du cordon en 2009 aux Etats-Unis. De même Bernard *et al.* [14] avaient trouvé une association significative entre la primiparité et la survenue de circulaire. Le post terme (42 SA) était significativement associé à la circulaire du cordon (Tableau 2). Ce résultat corrobore avec celui retrouvé par Shaffer *et al*. [4] où l’incidence des circulaires du cordon augmentait avec le terme. Par contre, Tahmakar *et al*. [15] avaient trouvé que la prématurité était un facteur de risque de la circulaire du cordon.

### Caractéristiques fœtales et funiculaires

La longueur du cordon =70 cm (Tableau 3) était un facteur de risque de circulaire du cordon dans notre étude. Dans les études antérieures, Rogers *et al.* en 2003 [16], Balkwade *et al*. en 2012 [17] et Adenesia *et al*. en 2014 [18] avaient montré que les nouveau-nés avec longue taille du cordon faisaient plus de circulaire. Le sexe masculin était un facteur de risque de circulaire dans notre étude (Tableau 2). Plusieurs études comme celles de Rhoades *et al*. en 1999 [13], Rogers *et al*. en 2003 [16] et Wang *et al*. en 2016 [19] ont retrouvé des résultats similaires avec parfois des fortes associations significatives entre le sexe masculin et la survenue de la circulaire du cordon. En effet, les fœtus de sexe masculin auraient plus de mouvements actifs fœtaux, ce qui les prédisposerait à faire plus de circulaire que ceux de sexe féminin [20]. Le poids des nouveau-nés de notre population d’étude (Tableau 3) n’avait pas d’association significative avec la survenue de circulaire du cordon. Ceci corrobore l’étude de Carey *et al.* en 2003 qui n’avaient retrouvé aucune association entre le poids du nouveau-né et la survenue de la circulaire du cordon [21].

### Régression logistique

Après régression logistique (Tableau 4), les facteurs de risque indépendants de circulaire du cordon étaient: l’âge gestationnel =42 SA, la longueur du cordon = 70cm, le sexe masculin du nouveau-né.

## Conclusion

La circulaire du cordon est fréquente. Les facteurs de risque indépendants peuvent être modifiables (post terme) ou non (longueur du cordon, sexe du nouveau-né). Nous suggérons aux décideurs d’améliorer le plateau technique des structures hospitalières afin d’améliorer les techniques de diagnostic anténatal de circulaire du cordon, de former le personnel de santé afin de diagnostiquer tôt et mieux prendre en charge les nouveau-nés avec circulaire du cordon. Les cliniciens devraient mieux surveiller les grossesses afin de prévenir les différentes complications liées aux circulaires du cordon et éviter autant que possible le post terme.

### Etat des connaissances actuelles sur le sujet

- Au Cameroun, le taux de mortalité périnatale lié au circulaire du cordon varie de 6,1% à 6,8%;
- La circulaire du cordon représente l’anomalie la plus fréquente du cordon et peut affecter toutes les grossesses;
- Le diagnostic anténatal de circulaire du cordon est surtout basé sur l’échographie.

### Contribution de notre étude à la connaissance

- Un peu plus de 15% (15,15%) de nouveau-nés issus des grossesses mono fœtales naissent avec une circulaire de cordon;
- La quasi-totalité des circulaires se retrouve autour du cou;
- L’âge gestationnel ≥ 42 SA, la longueur du cordon ≥ 70cm, le sexe masculin du nouveau-né sont indépendamment associés au circulaire du cordon.

## Remerciements

Nous remercions les administrations de l’HCY, du CHU et du CASS de Nkoldongo pour avoir donné l’autorisation de réaliser cette étude, ainsi que le personnel des unités de gynécologie et obstétriques et les parturientes pour avoir facilité sa réalisation.
